# The importance of informal support and a listening ear: A narrative analysis of emotional conflict and support needs of siblings of children with a psychiatric disorder

**DOI:** 10.1177/13591045221127929

**Published:** 2022-09-20

**Authors:** Elien E Veldhuizen, Gaston Franssen, Patricia F Schothorst, Floortje E Scheepers

**Affiliations:** 1 General Practitioner, 8125 Utrecht University, Utrecht, The Netherlands; 2 Humanities, 1234 University of Amsterdam, Amsterdam, The Netherlands; 3 University Medical Centre Utrecht Psychiatry, Utrecht, The Netherlands

**Keywords:** hermeneutic phenomenology, psychiatric disorder, narrative research, sibling support, personal experiences, emotional conflict, support needs, siblings, children

## Abstract

This study investigates the self-reported impact of children’s psychiatric disorders on their siblings and assesses what forms of support such children most value. We used a qualitative research design with open interviews to stimulate children between 8 and 15 years old to talk about their experiences living with a brother or sister with a psychiatric disorder. Their stories were analysed within a hermeneutic phenomenological framework in order to identify narrative themes and interpret the meaning of shared experiences. From our analysis, nine shared narrative themes emerge. Overall, siblings report feeling conflicted about adapting their lives to their brother’s or sister’s disorder and signal a need for personalized attention from parents. They also indicate that being involved in the care for their brother or sister helps them to better understand their behaviour. Finally, siblings reveal that, in their experience, formal, protocolized forms of support foreground family problems and stress. Thus, we recommend to involve children in the care process; to acknowledge their personal needs and conflicts; and to be mindful of the style of support: help offered in an informal or playful way, instead of formal and protocolized, could be a more effective way of meeting siblings’ needs.

## Introduction

Worldwide around 13.4% of all children and adolescents have psychiatric disorders (Guilherme et al., 2015) and many of them grow up with siblings. Growing up with a brother or sister with a psychiatric disorder can have important consequences for children: the sibling relationship has a major influence on personal development of brothers and sisters (Boer, 2012; Dijken, 2013) – in a negative as well as a positive sense. On the one hand, the undiagnosed siblings are at risk of also developing mental problems resulting from adversities in their social functioning, school performance or psychological well-being (Boer, 2012; Mascha & Boucher, 2006; Naylor & Prescott, 2004; Pit-Ten Cate, 2000; Verhulst, 2015). Recent research affirms an increased prevalence of psychopathology in siblings of children with mental health problems (Ma et al., 2020); in a similar vein, growing up with a brother or sister with a developmental disorder may affect siblings’ empathy skills and theory of mind ability (Eyuboglu et al., 2018). On the other hand, living with a brother or sister with a psychiatric disorder can also result in increased maturity, improved patience, and better social skills (Meadan et al., 2010; Okma et al., 2015).

It is generally agreed upon that siblings of children with a psychiatric disorder benefit from additional support (Cox et al., 2003; Moyson & Roeyers, 2012). Thankfully, several options for help have already been identified. Parents, first and foremost, play an important role for guidance and support (Boer, 2012; Okma et al., 2015). Support from other family members can further ‘buffer’ the impact of living with a child with a psychiatric disorder (Giallo & Gavidia-Payne, 2006). There is also a (limited) choice of professional help, such as support groups and training. Although there is a growing body of literature on effects of sibling support groups in a psychiatric context, these studies often focus on specific conditions (Jones et al., 2020; Tudor & Lerner, 2015). Additionally, these researchers emphasize that more research is required to best address the needs of siblings.

Existing research investigating the consequences for siblings of children with a psychiatric disorder has been mainly concerned with children with an autism spectrum disorder (Eyuboglu et al., 2018; Mascha & Boucher, 2006). More importantly, most research focuses on reports by parents and does not acknowledge the siblings’ own perspectives (Carel & Györffy, 2014; Meadan et al., 2010). An exception is the work of Moyson & Roeyers, who examined personal stories of siblings by conducting in-depth interviews in order to assess their quality of life (Moyson & Roeyers, 2012). The authors indicate that analysis of such stories can help to meet the needs of siblings.

Despite existing insights in the effects of living with a child with a psychiatric diagnosis on its siblings, then, little is known about what the personal stories of the latter indicate about *their* experiences, what *they* require, and how researchers and health-professionals can benefit from such stories. The objective of this research, therefore, is to acknowledge the perspectives of children with a brother or sister with a psychiatric disorder by focusing on their self-reported experiences and needs. Our aim is to offer a qualitative analysis of stories of children with a diagnosed brother or sisters and to trace thematic patterns that emerge in these stories. Our study is part of the Stories Database Psychiatry project (Verhalenbank Psychiatrie) of the Utrecht University Medical Centre, in which personal narratives of psychiatric patients, relatives and professionals are collected and analysed with the aim to improve patient care (Verhalenbank, n.d.). In this study, we collected narratives of siblings of children with a psychiatric disorder in order to assess the impact of having an affected brother or sister. There is growing evidence that young children are indeed able to report accurately on their experiences if given the opportunity and when using questions at their level of understanding (Moyson & Roeyers, 2012). Accordingly, the goal of our research is to explore which forms of support siblings point out to be helpful (or unhelpful) to them and why, so that forms of support might be best aligned with their needs.

## Method

### Participants

We included individuals through purposive sampling, as we wanted to gain knowledge about comparable experiences related to specific psychiatric and family-related conditions. Therefore, we only included children between 8 and 16 years, with a brother or sister diagnosed with a psychiatric disorder and younger than 18 years. Participants were recruited between March 2017 and March 2018 in two ways: written invitations were sent to parents from children hospitalized in the child psychiatric day clinic of the University Medical Centre Utrecht; and information folders were presented to parents attending lectures on developmental disorders organised by the same institution in 2017.

If siblings were diagnosed with a psychiatric disorder themselves, they were excluded. The specific diagnoses of the brothers or sisters of these children were not always unproblematically comparable in themselves, as there are distinct differences between, for instance, ADHD and PDD NOS, but as our interest was with the communalities in the siblings’ experiences, we included children with brothers or sisters diagnosed along a spectrum of psychiatric disorders.

Finally, 13 siblings, belonging to 12 families, were included with an average age of 10.5 years and a distribution of 8–15 years: 6 female participants and 7 male. The average age of the 15 affected children was 11.3 years and 80% was male. The sample size may be small, but with relatively homogeneous groups like this, data saturation can occur between 6 to 12 interviews (Guest et al., 2006). Demographics and characteristics of the affected children are shown in Table 1. This project was approved by the Medical Ethical commission of the University Medical Center Utrecht; written informed consent was obtained from participants and both their parents.

**Table 1.** Characteristics of the children with a psychiatric disorder.

Variable	(Affected) children (*n* = 15)^*^
Age of child, average (distribution)	11.3 (7–17)
Gender, n (%)	
Male	12 (80%)
Female	3 (20%)
Type of psychiatric diagnosis	
Developmental disorder	12 (80%)
ADHD	
ASS (PDD NOS, asperger, autism)	
Unspecified disruptive, impulse-control, and conduct disorder	2 (13%)
Depressive disorder	1 (7%)
Intellectual disability	1 (7%)

*Three participants had two brothers/sisters with a psychiatric disorder, two participants came from the same family with one child with a psychiatric disorder.

### Data collection

We conducted in-depth, unstructured interviews with the participants in Dutch. We chose a qualitative research design, as described below. Several measures were taken to create a feeling of trust with the participants, as this is essential when interviewing children (Moyson & Roeyers, 2012). Siblings were given the opportunity to choose an interview location where they would feel most at ease. Furthermore, an open topic list was used, giving participants the opportunity to suggest their own topics. Interview started with open, personal questions (e.g. ‘What makes you happy?’, ‘What are your wishes?’) so that children felt at ease before talking about their relationship with their brother or sister. Interviews lasted a maximum of 60 minutes. The interviews date from 2018 as that this project was carried out in the context of (voluntary) internships and is quite time-consuming by nature. Nonetheless, it is safe to assume that the collected data is still relevant, as there have been no fundamental changes to the diagnosed condition or to the health-care context.

### Data processing and analysis

Interviews were recorded and transcribed by medical students. The transcripts were analysed in a qualitative method based on hermeneutic phenomenology. Hermeneutic phenomenology is a commonly used method to interpret the meaning of interviewees’ experiences It aims at at identifying aspects of subjective experiences as well as evaluating and interpreting their meaning (Lindseth & Norberg, 2004; Manen, 2005; Ricoeur, 1976). Our assumption, then, is that an hermeneutic-phenomenological analysis of personal stories offers access to how individuals experience and evaluate life conditions. By singling out aspects of experiences as recounted in specific sections of a participant’s narrative and comparing them to related experiences in other sections of the same participant’s narrative, and subsequently to experiences as recounted by *other* participants, recurring and shared ‘themes’ begin to emerge. This way, the individual narrative can be analysed as offering insights into shared experiences. Previously, this method has been used to study narratives of parents with children with a psychiatric disorder (Pejlert, 2001) and to interpret experiences of siblings of children with autism and intellectual disability (Moyson & Roeyers, 2012).

**Figure 1.**
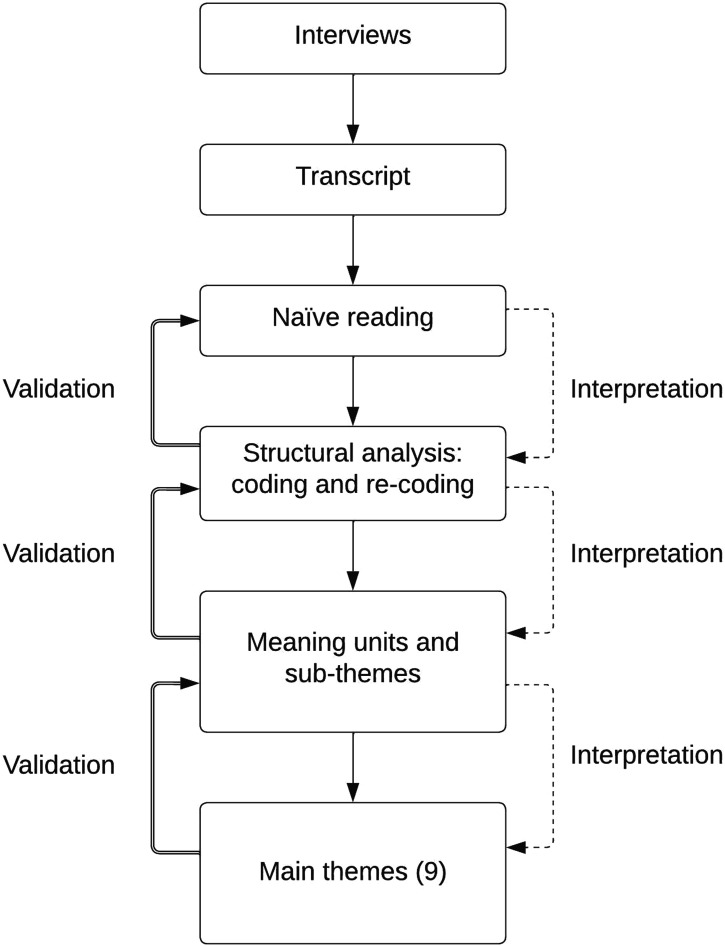
Data collection and analysis.

Our analysis consisted of three phases. First, an initial or ‘naïve’ reading of the transcripts resulted in a preliminary understanding of the overall narrative. Secondly, recurring structures were identified and coded. The text of the transcript was subsequently divided into ‘meaning units’ – text parts revolving around specific experiences. By comparing the (individual) ‘sub-themes’ in the meaning units amongst the different interviews, overarching, shared ‘main themes’ could be identified. Thirdly, all meaning units, subthemes and main themes where continuously validated against the background of the results of the previous analytic phases. This resulted in a circular process (see Figure 1) that ensured that the findings of each stage of the analysis aligned with the previous stages. After a first full analysis, the text was read by a second assessor. All analyses and codes were discussed amongst the research group members.

## Interpretation and results

The *naïve reading* resulted in a general understanding of the overall structure and content of the narrative. In most stories, siblings mentioned the difficulties in living together with their brother or sister with a psychiatric disorder, emphasizing their struggles to deal with conflicts and unpredictable behaviour. They mentioned positive experiences too, as well as coping mechanisms, but the emphasis was on difficulties and challenges. Notably, the stories expressed feelings of ambivalence: the siblings expressed their appreciation of growing up together, but also indicated that this required continuous adjustment, as they felt that they needed to prioritize their brother or sister’s needs. The overall message of their stories was that siblings require attention and support from loves ones or relatives and do not necessarily value professional help.

The *first structural analysis* focused on narrative categories (e.g. metaphors, common stop words, protagonists and temporal perspectives) (Peljert, 2001). The siblings and their brother or sister were the stories’ protagonists. Co-actors were parents or other family members. The temporal perspective was mostly the present. The future tense was mostly used to describe hopes and wishes for a positive sibling relationship. Occasionally, siblings talked about the past to describe the development of their relation with their affected brother or sister. Most participants preferred the use of first-person, but some switched to second-person when discussing difficult situations or expressing wishes and desires. Participants used vague expressions such as ‘or so’ and ‘etcetera’ when describing disrupting behaviour, their parents’ approach to it or their own feelings related to it. Some younger participants gave evading answers or did not reply to specific questions, mainly when talking about their own (negative) feelings or experiences directly related to their brother or sister:

‘Do you find it difficult to talk about R.?’... ‘Yes, I think that is kind of difficult... [Silence]... Uhm, I am not very used to it yet.’ (E.,8 years old)

In the *second structured analysis* all transcripts were coded with meaning units that captured recurring experiences. This resulted in sub-themes that capture returning and shared stories about events (e.g. ‘Disrupting behaviour’), emotional impact (e.g. ‘Processing difficult feelings’), or impact (‘Influence on everyday choices’).

To achieve a deeper understanding of the stories, the *third structured analysis* focused on the story as a whole, comparing the naïve reading with the identified sub-themes. Clustering the sub-themes made it possible to identify nine ‘main themes’. All main themes and sub-themes with example quotes can be found in Table 2. In addition, we provide general clarifying remarks on the main themes below. We acknowledge that it may appear somewhat counterintuitive for a phenomenology-based analysis to abstract siblings’ responses from their storied context; our goal, however, was to chart the shared, supra-individual experiences of living with a diagnosed brother or sister.

**Table 2.** Main themes and sub-themes.

Main themes	Sub-themes	Example quotes
1. Conflicts	Physical aggression; Verbal aggression; Disruptive behaviour; Recurring fights; Seclusion; Inaccessibility	‘Almost every time I play with my brothers, one of them gets into a fight. And then it does not stop anymore... For example, mom and dad have given many examples how to stop quarrels, but it just doesn’t help.’ (J.,8 years old) ‘My sisters are mostly on their computer, or just on their phone. And then they are really in their own world. It becomes very difficult to communicate with them because they are so much into their own game.’ (Y.,14 years old)
Coping strategies	Accepting behaviour and adjusting (own) behaviour Anticipating needs or potential conflicts; Leave sibling alone Ignoring disturbing behaviour Avoiding actions Impulsive reactions Seeking support Positive, calm attitude	‘My whole life, I have always taken into account my sisters’ situation. So I do not always realize that I take it into account, because it has just become automatic...’ (Y.,14 years) ‘When my brother is angry then I just say to him “can you maybe stop hitting and kicking me” and I say that all the time and then finally he will stop... But if he doesn’t stop, then I just run away, like around the room, and I go and tell mom…’ (K.,8 years) ‘Sometimes all the attention goes to R., and then I think like “yeah, I do not longer exist”... [Silence] then I just go to my room, as [my parents] are now solely focused on my brother. They don’t know that I am in my room. They don’t know that I am very sad...’ (E.,8 years)
Need for rest and time alone	Not feeling like adjusting; Time alone/private time with parents	‘I like to draw... Then I am away from my world for a while, then I can finally do something for myself.’ (E.,8 years) ‘Q. asks me always in the weekends “do you want to play with me on the PlayStation?” I usually say yes, but sometime I say no... It is sometimes difficult to say no... Sometimes I would just like to play by myself…’ (S.,10 years)
Need for support and attention	Talking to parents; Talking to other significant persons; Finding it difficult not to be able to go to parents; Reluctance towards professional help	‘Then he sometimes starts to shout and he hurts me. I do not like that. Then I usually go to mommy or I go to my room... Mommy always makes me happy again and then I will do something with mommy, for example...’ (J.,10 years) ‘I usually go to my teacher when I feel sad... We talk about many things, how things are going at home. And sometimes about how I can better concentrate, so that I do not get stuck all the time in the feeling that I no longer exist.’ (E.,8 years) ‘Well, I would not really need support. They tried this in the beginning... Well, I didn’t like it at all... A lady started to explain me all kinds of things how I could handle things better... It was a nice lady, though. But this was not really my thing. I just prefer to decide for myself how I handle things.’ (C.,14 years) ‘Sometimes there is too much help from the psychologist for me and I don’t like that at all. Then all the attention goes to me again and not even once to my brother..’ (E.,8 years) ‘I quite liked the support group, because there are also other children who have this [experience], so I am not the only one who has such a brother who is always angry...’ (K.,8 years)
Wish for normality	Fantasy about life without sibling or with different brother/sister	‘My wish is that my brother would be normal... Because than I could be much better at playing soccer, because my friend has a brother who is very good at playing soccer, so his brother taught him that...’(T.,8 years) ‘It would be very different to have no brothers or sisters... Sometimes I just think, why do they exist?’ (J.,8 years)
Influence on personal choices and possibilities for development	Influence on everyday choices; Influence on choices for the future;Influence on personal development	‘When my sisters would not have had autism, I think I would have been a different person... Then I would have had a completely different life. Then my personality would have been different too, because now I am very organised.’ (Y.,14 years) ‘When she is at home, a lot of attention goes to her… For example, if we go to my father, they mainly look mainly at my sister, like “what does she want?” and: “at what time should she be back at home?”... I do not really have a say in that. I do understand it as well, but of course I also find it a bit annoying that I am not allowed to decide for myself when I want to go home, for example, or how late...’ (M.,15 years)
Doing things together	Paying attention to needs of brother/sister;Being dependent on behaviour;Appreciating sharing of positive experiences	‘It makes me happy to play with S.. On holidays, we go swimming together...’ (T.,8 years) ‘When he is sweet, he gives me hugs and such. And then we also play board games together.’ (K.,8 years) ‘I would like T. to be nicer. And that T. will play less computer games. So that we will be able to play together more often.’ (J.,10 years)
Recommendations and advice	Clarity in communication;Positivity and tranquillity Ignoring disturbing behaviour or not taking it personally Approaching fights in a playful way Seeking help from parents	‘You should especially say clearly to the person what he or she really needs to do or should do. You should stay calm and try not to panic.’ (S.,10 years) ‘Because when T. said something ugly, it often made me very sad... To not take it personally and say to yourself in your head “I do not take it personally”.’ (J.,10 years) ‘I had to write on a note or something about what kind of negative things I said to him and what kind of negative things he said to me. That worked quite well, because then you start to pay attention to it and it becomes much less...’ (A.,14 years)
Ambivalence and loyalty	Ambivalent feelings;Loyalty to parents and brother/sister	‘Sometimes I like it that R. is somewhere else, but it can also be difficult for me... That he is not annoying me all the time, and then I also have time for myself... But then I also miss him very much.’ (E.,8 years) ‘Sometimes S. does get more attention, but I don’t mind at all because S. is different, so that’s part of the situation...’ (T.,8 years)

In sum, we found sub-themes that could be clustered into nine main themes related to experiences of ‘Conflict’ (e.g. ‘Physical aggression’), descriptions of ‘Coping strategies’ (e.g. ‘Leave sibling alone’), indications of a ‘Need for rest and time alone’ as well as a ‘Need for support and attention’ (e.g. ‘Talking to parents’), expressions of a ‘Wish for normality’ (‘Fantasy about life without sibling or with unaffected brother or sister’), descriptions of the ‘Influence on personal choices’ (e.g. ‘Influence on everyday choices’), indications of the importance of ‘Doing things together’ (e.g. ‘Appreciating sharing of positive experiences’) and the children’s own ‘Recommendations and advice’ (e.g. ‘Clarity in communication’).

Together, these themes indicate the narrative shared by the siblings: how they experience living with an affected brother or sister, what needs they have, and in which ways these needs can be best addressed. In all stories siblings mentioned conflicts, often involving verbal or physical aggression. These confrontations cause negative emotions. Siblings show a remarkable level of reflection on how to avoid or solve conflicts. They attempt to adjust themselves in behavioural, cognitive and emotional ways. Siblings are able to reflect on coping strategies: these include seeking support from parents, ignoring the difficult behaviour, or adjusting their behaviour (see Table 2).

Siblings express a need for time alone and private activities with their parents. They acknowledged attempts to treat all children equally, but find that their parents are not always successful in doing so. However, they realize and accept that their brother or sister simply needs more care. Since they feel loyal to their family members, they are willing to adjust.

To be able to talk about their problems and emotions is very important for siblings in order to feel supported. They prefer to talk to their parents, but also accept support from other significant persons. Interestingly, they generally do not indicate a specific need for professional help, such as a parent mentor or psychologist: they would rather play than have a formal conversation. Additionally, they mention having had positive experiences with sibling support groups, in which help is offered in a playful way, for instance through a game. Advice that they would offer other siblings is to maintain a patient and calm attitude, to communicate clearly or to ignore disrupting behaviour (see Table 2).

Sometimes, siblings fantasize about what their live would be like without their brother or sister. They then come to reflect on how their brother or sister influences their lives, both in positive and negative ways. Overall, the stories demonstrate a certain longing for ‘normality', for a way of living that does *not* foreground that their family situation is ‘different’ and problematic.

## Discussion

In this study, we found that siblings of children with a psychiatric disorder were able to describe and evaluate their experiences: they showed a high level of reflection on the impact of living together with an affected brother or sister.

The interviews created a safe space for detailed exploration of the siblings’ experiences. From their stories, nine main themes emerged. Some of the themes are confirmed by previous research: these include the need for private time and sharing or exchanging experiences with others (Moyson & Roeyers, 2012).

Additionally, the narratives revealed that living with an affected brother or sister has a profound impact on siblings. They experience ambivalent feelings towards their brother or sister and find themselves in an emotional conflict. They love their brother or sister, but they struggle with their disruptive behaviour. Simultaneously, they feel guilty about having feelings of frustration and anger. The narratives indicate that this emotional conflict often results in strategic adaptation of behaviour to maintain stability and avoid stress. Consequently, as described in previous research (Cox et al., 2003; Ross & Cuskelly, 2006), the siblings develop a variety of coping strategies. Paradoxically, the need for a ‘normal’ and uncomplicated family life requires ‘artificial’ adaptation from the siblings and intense reflection on the interaction with their brother or sister.

Being used to adapting to their brother or sister, and pleasing their parents, the siblings often ignore their own needs, which can lead to negative emotions. Unfortunately, in choosing family activities, the wishes of the affected child tend to be prioritized. Consequently, siblings miss private time and undivided attention (Hastings, 2014).

Finally, this research leads us to formulate four recommendations to better meet siblings’ needs. First, it is important to stimulate siblings to talk about their feelings. It is well-known that when children have an understanding of their family situation, they will be more effective in processing their experiences and expressing their needs (Boer, 2012). However, we found that siblings were often not used to talk about feelings. They struggled with describing challenging situations and negative emotions related to their affected brother or sister. Talking about these experiences can cause stress or anxiety (Moyson & Roeyers, 2012). Additionally, they are used to deprioritize themselves (Okma et al., 2015). They come to ignore their own emotions, with a risk of developing mental problems (Garley & Johnson, 1994). Our advice to offer a listening ear to siblings of children with a psychiatric disorder, then, applies in the first place to the parents and relatives. In addition, health care professionals as well as other people involved should give siblings opportunities to talk about their feelings and should acknowledge their needs.

Second, we recommend to involve siblings in their brother or sister’s treatment and offer them relevant psychoeducation. Previous research has shown that siblings find it easier to accept the disorder of their brother or sister if they have a better understanding of it (Garley & Johnson, 1994; Moyson & Roeyers, 2012). When siblings know how to deal with the situation, they tend to experience a better quality of life. Simple tips on how to respond to disrupting behaviour can already contribute to a less frustrating and more effective way of communicating. It is also important to undertake joined activities, as these have a positive effect on the brother-sister relationship (Boer, 2012; Moyson & Roeyers, 2012).

Third, it is important to offer close, dedicated support from parents or caregivers. Even though other significant persons are mentioned (e.g. extended family members, friends), siblings point out that they prefer talking to their parents and having one-on-one activities with them. Research shows that open communication between parents and children is an important preventive factor for the development of psychological problems in siblings (Moyson & Roeyers, 2012; Pit-Ten Cate, 2000), while insufficient availability of parents can lead to negative emotions (e.g. feelings of not being important enough) in children. Understandable, many parents experience difficulties adequately dividing the attention between their children (Delfos, 2009; Okma et al., 2015), but we found that some parents of the interviewed sibling admitted to underestimating the impact of the family situation on their non-diagnosed children. Thus, it is crucial that parents do not to underestimate the importance of being available for siblings.

Fourth, we suggest to be careful with respect to offering professional – that is, formerly ‘marked’ as professional – help, since this can be counterproductive when siblings experience too much attention and feel that their problem gets too ‘loaded’. Interviewed siblings did mention positive experiences with support groups focussing on sharing or exchanging expression through playful activities. Realizing that the problems they face are not unique, the siblings experience recognition, shared awareness and acknowledgement of their feelings (Evans et al., 2001; Naylor & Prescott, 2004; Okma et al., 2015). In general, it is important to give children the opportunity to play with each other and offer help in an informal way. This way, they can learn how to better process and appreciate their personal experiences (Moyson & Roeyers, 2012; Naylor & Prescott, 2004).

## Limitations of the study

The number of participants in this study was relatively small (*n* = 13) and in the affected siblings there was a variety of diagnoses. However, siblings’ responses to questions related to support needs were highly comparable. Additionally, some researchers argue that in smaller, preliminary, explorative studies, saturation – the point at which no new themes are observed in the data – can be achieved with eight to 10 participants (van Geelen et al., 2013). Another limitation was that participants signed up through their parents: some parents refused to allow their child to participate, resulting in possible selection bias. Additionally, we mainly talked to siblings of children with a developmental disorder or behaviour disorder so we missed other diagnosis like psychotic disorders or depression. Furthermore, there was a relatively wide age range of siblings (8–15 years) and the sample size was too small to compare the different age groups. Finally, all participants were recruited in the region of Utrecht, which could cause bias as the mental health system might be organized differently in other regions.

## Future research

The results invite follow-up research on the facilitation of support for siblings of children with a psychiatric disorder, as well on the role of family members and of professionals in the healthcare sector and social care. Since siblings’ support need may be interrelated with the characteristics of the disorder of their brother or sister, future research could focus on children with other disorders than those included here or in other research, e.g. depression or anxiety disorder. Additionally, children’s needs may be influenced by their developmental stage, cultural factors and socio-economic environment: all these are factors of interest for future research. Finally, the results of this study, in line with extant research literature, point out the possibly increased efficacy of professional help when offered in an informal or playful manner. Despite these hopeful results, more research is needed to ensure such interventions will be sufficiently valid and reliable.

## References

[bibr3-13591045221127929] Boer F . (2012). Broers en zussen van speciale en gewone kinderen : Invloed op ontwikkeling en gedrag. Lannoo Campus.

[bibr4-13591045221127929] Carel H. Györffy G. (2014). The art of medicine: Seen but not heard: Children and epistemic injustice. Lancet, 384(9950), 1256–1257. 10.1016/s0140-6736(14)61759-1 25289422

[bibr6-13591045221127929] Cox A. H. Marshall E. S. Mandleco B. Olsen S. F. (2003). Coping responses to daily life stressors of children who have a sibling with a disability. Journal of Family Nursing, 9(4), 397–413. 10.1177/1074840703258328

[bibr7-13591045221127929] Delfos M. Martine F. (2009). Ontwikkelingspsychopathologie: Stoornissen en belemmeringen. 7^e^ druk. Pearson.

[bibr8-13591045221127929] Dijken A. van. (2013). Broers en Zussen Boek- voor & door brussen met een bijzondere broer of zus. Lannoo Campus.

[bibr10-13591045221127929] Evans J. Jones J. Mansell I. (2001). Supporting siblings: Evaluation of support groups for brothers and sisters of children with learning disabilities and challenging behaviour. Journal of Learning Disabilities, 5(1), 69–78. 10.1177/146900470100500107

[bibr11-13591045221127929] Eyuboglu M. Baykara B. Eyuboglu D. (2018). Broad autism phenotype: Theory of mind and empathy skills in unaffected siblings of children with autism spectrum disorder. Psychiatry and Clinical Psychopharmacology, 28(1), 36–42. 10.1080/24750573.2017.1379714

[bibr12-13591045221127929] Garley D. Johnson B. (1994). Siblings and eating disorders: A phenomenological perspective. Journal of Psychiatric and Mental Health Nursing, 1(3), 157–164. 10.1111/j.1365-2850.1994.tb00039.x 15835316

[bibr13-13591045221127929] Giallo R. Gavidia-Payne S. (2006). Child, parent and family factors as predictors of adjustment for siblings of children with a disability. Journal of Intellectual Disability Research, 50(12), 937–948. 10.1111/j.1365-2788.2006.00928.x 17100954

[bibr14-13591045221127929] Guest G. Bunce A. Johnson L. (2006). How many interviews are enough? An experiment with data saturation and variability. Field Methods, 18(1), 59–82. 10.1177/1525822x05279903

[bibr15-13591045221127929] Guilherme V. Giovanni A. S. Luisa S. S. Arthur C. Luis A. R. (2015). Annual research review: A meta-analysis of the wordwide prevalence of mental disorders in children and adolescents. The Journal of Child Psychology and Psychiatry, 56(3), 345–365. 25649325 10.1111/jcpp.12381

[bibr16-13591045221127929] Hastings R. (2014). Children and adolescents who are the siblings of children with intellectual disabilities or autism: Research evidence. Young Siblings Evidence Review, 1–11.

[bibr17-13591045221127929] Jones E. A. Fiani T. Stewart J. L. Neil N. McHugh S. Fienup D. M. (2020). Randomized controlled trial of a sibling support group: Mental health outcomes for siblings of children with autism. Autism, 24(6), 1468–1481. 10.1177/1362361320908979 32169003

[bibr18-13591045221127929] Lindseth A. Norberg A. (2004). A phenomenological hermeneutical method for researching lived experience. Scandinavian Journal of Caring Sciences, 18(2), 145–153. 10.1111/j.1471-6712.2004.00258.x 15147477

[bibr19-13591045221127929] Ma N. Roberts R. Winefield H. Furber G. , 2020. A dimensional approach to the mental health of siblings of children with mental health problems: A 20-year systematic review. Journal of Family Studies, 26(2), 308–328. 10.1080/13229400.2017.1375966

[bibr20-13591045221127929] Manen M. Van. (2005). Fenomenologie: Een kwalitatieve stroming met een verscheidenheid aan tradities. Kwalon, 10(1), 30–36.

[bibr21-13591045221127929] Mascha K. Boucher J. (2006). Preliminary investigation of a qualitative method of examining siblings’ experiences of living with a child with ASD. The British Journal of Development Disabilities, 52(102), 19–28. 10.1179/096979506799103659

[bibr22-13591045221127929] Meadan H. Stoner J. B. Angell M. E. (2010). Review of literature related to the social, emotional, and behavioral adjustment of siblings of individuals with autism spectrum disorder. Journal of Developmental and Physical Disabilities, 22 (1), 83–100. 10.1007/s10882-009-9171-7

[bibr24-13591045221127929] Moyson T. Roeyers H. (2012). “The overall quality of my life as a sibling is all right, but of course, it could always be better”. Quality of life of siblings of children with intellectual disability: The siblings’ perspectives. Journal of Intellectual Disability Research, 56(1), 87–101. 10.1111/j.1365-2788.2011.01393.x 21366753

[bibr25-13591045221127929] Naylor A. Prescott P. (2004). Invisible children? The need for support groups for siblings of disabled children. British Journal of Special Education, 31(4), 199–206. 10.1111/j.0952-3383.2004.00355.x

[bibr26-13591045221127929] Okma K. Naafs L. Vergeer M. Dijken van A. (2015). *QuickScan naar de ondersteuningsbehoefte van zorgintensieve gezinnen*. Visiondocument part 2: Siblings. The Dutch Youth Institute.

[bibr27-13591045221127929] Pejlert A. (2001). Being a parent of an adult son or daughter with severe mental illness receiving professional care: Parents’ narratives. Health and Social Care in the Community, 9(4), 194–204. 10.1046/j.0966-0410.2001.00301.x 11560735

[bibr28-13591045221127929] Pit-Ten Cate G. M. P. (Ineke) L, I. (2000). Experiences of siblings of children with physical disabilities: An empirical investigation. Disability and Rehabilitation, 22(9), 399–408. 10.1080/096382800406013 10894203

[bibr29-13591045221127929] Ricoeur P. (1976). Language as discourse. In Interpretation theory: Discourse and the surplus of meaning (pp. 1–22).

[bibr30-13591045221127929] Ross P. Cuskelly M. (2006). Adjustment, sibling problems and coping strategies of brothers and sisters of children with autistic spectrum disorder. Journal of Intellectual & Developmental Disability, 31(2), 77–86. 10.1080/13668250600710864 16782592

[bibr31-13591045221127929] Tudor M. E. Lerner M. D. (2015). Intervention and support for siblings of youth with developmental disabilities: A systematic review. Clinical Child and Family Psychology Review, 18(1), 1–23. 10.1007/s10567-014-0175-1 25315924

[bibr33-13591045221127929] van Geelen S. M. Bolt I. L. E. van der Baan-Slootweg O. H. van Summeren M. J. H. (2013). The controversy over pediatric bariatric surgery: An explorative study on attitudes and normative beliefs of specialists, parents, and adolescents with obesity. Journal of Bioethical Inquiry, 10(2), 227–237. 10.1007/s11673-013-9440-0 23585016

[bibr34-13591045221127929] Verhalenbank Psychiatrie Verhalenbank Psychiatrie | Verhalenbank Psychiatrie . (n.d.). http://psychiatrieverhalenbank.nl/

[bibr35-13591045221127929] Verhulst F. C. (2015). Leerboek Kinder- en Jeugdpsychiatrie. 4^e^ herziende druk *Koninklijke van Gorcum*.

